# Uptake of prostate cancer screening and associated factors among Chinese men aged 50 or more: a population-based survey

**DOI:** 10.7497/j.issn.2095-3941.2014.01.005

**Published:** 2014-03

**Authors:** Winnie K.W. So, Kai Chow Choi, Winnie P.Y. Tang, Paul C.W. Lee, Ann T.Y. Shiu, Simone S.M. Ho, Helen Y.L. Chan, Wendy W.T. Lam, William B. Goggins, Carmen W.H. Chan

**Affiliations:** ^1^The Nethersole School of Nursing, The Chinese University of Hong Kong, Hong Kong SAR, China; ^2^Department of Community Medicine, The University of Hong Kong, Hong Kong SAR, China; ^3^The Jockey Club School of Public Health and Primary Care, The Chinese University of Hong Kong, Hong Kong SAR, China

**Keywords:** Prostatic neoplasms, early detection of cancer, prostate-specific antigen (PSA), population-based survey

## Abstract

**Objective:**

To investigate the uptake rate of prostate specific antigen (PSA) testing among Hong Kong Chinese males aged 50 or above, and identify factors associated with the likelihood of undergoing a PSA test.

**Methods:**

A population-based telephone survey was conducted in Hong Kong in 2007. The survey covered demographic information, perceived health status, use of complementary therapy, cancer screening behavior, perceived susceptibility to cancer and family history of cancer. Descriptive statistics, percentages and logistic regression analysis were used for data analysis.

**Results:**

A total of 1,002 men aged 50 or above took part in the study (response rate =67%), and the uptake rate of PSA testing was found to be 10%. Employment status, use of complementary therapy, perceiving regular visits to a doctor as good for health and the recommendations of health professionals were significant factors associated with PSA testing.

**Conclusion:**

The uptake rate of PSA testing in the study population was very low. Among all the factors identified, recommendations from health professionals had the strongest association with the uptake of PSA testing, and they should therefore take an active role in educating this population about cancer prevention and detection.

## Introduction

Globally, prostate cancer has been reported to be the second most common cancer and the sixth leading cause of death among men^1^. Compared with worldwide figures, the incidence of prostate cancer in China is low, estimated in 2008 to be more than 33,000 with the age-standardized incidence and mortality rate of 4.3 and 1.8 per 100,000, respectively. However, the trend is increasing, and it is estimated that by 2020 new cases will number over 49,000 and account for more than 20,000 deaths^2^. Locally, the incidence of prostate cancer has also been increasing over the past two decades. In 2010, prostate cancer was the third most common cancer and the fifth leading cause of cancer death in males. The Hong Kong Cancer Registry^3^ reported in 2010 that there were 1,492 new cases, the age-standardized incidence rate was 28.1, and the age-standardized death rate was 5.5 per 100,000 of the standard population.

Although the causes of prostate cancer are not yet fully understood, it is believed that advanced age (above 50), a positive family history of prostate cancer and an African-American ethnic background are risk factors^4^^,^^5^. Early detection is commonly effected by serum prostate specific antigen (PSA) screening, digital rectal examination (DRE) and transrectal ultrasound (TRUS). Among these three, PSA was found to be the most accurate single diagnostic tool, and more cost-effective than DRE or a combination of DRE and PSA^6^^,^^7^. The detection rate of DRE also depends on the level of PSA and the experience and technique of the physician. With low levels of PSA, the sensitivity of DRE was low, and its performance in detecting small-volume tumors was also poorer than PSA^8^.

However, the recommendation in early prostate cancer screening of serum PSA testing remains controversial. Arguments against such screening concern over-diagnosis, over-treatment and its consequent adverse effects^9^^,^^10^. One systematic review and meta-analysis found that the effect of screening on death and overall mortality from prostate cancer was insignificant^11^. In contrast, a large randomized controlled trial conducted by the European Randomized Study of Screening for Prostate Cancer (ERSPC) reported a 20% reduction in prostate cancer deaths^12^, and another observational study also found a significant decrease in such mortality^13^. PSA screening is also associated with an increased diagnosis of early stage prostate cancer and a decrease in advanced stage and distant metastases, and thus with higher survival rates^8^^,^^11^. Nevertheless, the American Cancer Society (ACS) recommends that men who are asymptomatic and have a life expectancy of at least ten years should discuss the matter with their physician and reach an informed decision about prostate cancer screening, with the pros and cons of such screening being fully explained. For men at risk, the optimal age for receiving such information depend on the level of risk—the higher the risk, the younger the age shared and informed decision-making should be initiated^5^. As the population is generally aging and the general public is more concerned about health, the need for screening is clearly increasing.

The uptake rates for prostate cancer screening among African Americans and Canadians were 36% and 47.5%, respectively^14^^,^^15^. In Chinese populations, the rate is low. In Taiwan, studies have reported the uptake rate to be between 12.4% and 29.4%^16^^,^^17^. Different studies have been conducted to investigate the factors associated with participation in prostate cancer screening. Qualitative studies have found fear of cancer, lack of knowledge, embarrassment and perceived low risk were barriers, while a family history of prostate cancer, urinary symptoms and physician’s recommendations were facilitators^18^^,^^19^. Age, educational attainment, household income, insurance coverage and marital status are common predictive factors^14^^,^^20^^,^^21^. Other contributing factors include chronic illness, obesity, medical visits and perceptions of health^14^^,^^20^^,^^21^.

Several studies have also investigated the facilitators and barriers in Chinese populations, with similar findings: physician’s recommendations, history of benign prostate hypertrophy, embarrassment, lack of knowledge, being asymptomatic, and financial status all being noted^16^^,^^17^. However, no study has been conducted in Hong Kong to investigate the rate of prostate cancer screening and the associated factors affecting uptake. In addition, even though Hong Kong and Taiwan share the same cultural origins, diverse sub-cultures have developed with the differences in historical backgrounds and geographical positions. Furthermore, there are differences in the social, political and economic systems between the two places^22^^,^^23^, which may cause variations in social norms and health-seeking behavior in their respective populations. It is therefore worth investigating the factors affecting prostate cancer screening in Hong Kong. Thus, the study aimed to investigate the uptake rate of PSA testing among Hong Kong Chinese males aged over 50, and identify the factors associated with the probability of that population undergoing PSA testing.

## Materials and methods

This study was conducted in 2007 using a population-based telephone survey. The methodology of the survey was described elsewhere^24^. In brief, participants of the present study were Hong Kong Chinese residents aged 50 years or above. A structured questionnaire consisted of six sections: demographics, perceived health condition, use of complementary therapy, cancer screening behaviour, perceived susceptibility to cancer and family history of cancer. In the section on complementary therapy, respondents were given a four-point rating scale to indicate how frequently they used it, where 0, never; 1, once only; 2, occasionally; 3, on a regular basis; and the 2005 cancer module of the National Health Interview Survey^25^ was used, in modified form, to collect data in the cancer screening behavior section. Ethical approval was obtained from the Survey and Behavioral Research Ethics Committee of the Chinese University of Hong Kong. Verbal consent was obtained from all participants. Telephone numbers were randomly selected from the updated residential directories, which include over 95% of households in Hong Kong, and anonymous interviews were carried out by the telephone survey team of the university’s Centre for Epidemiology and Biostatistics. Data collection went on from 6:30 pm to 10:30 pm only, to minimize over-representation of the non-working population. If there were two or more eligible household members, the member with birthday nearest to the interview date was recruited. To minimize any bias from non-responses, at least three calls were made at different times on the other days before assigning a non-response status to a number.

Data were entered and analyzed with SPSS 17.0 (SPSS Inc, Chicago, USA). Descriptive statistics was used to summarize and present the data. Frequencies (percentages) were used to present the results of categorical variables. Stepwise multivariable logistic regression analysis was used to identify factors independently associated with prostate cancer screening behavior, and the factors to be explored are listed in **Tables 1** and **2**. A binary outcome variable was used to study screening behavior: ‘Have you ever had a prostate-specific antigen (PSA) test?’ (Yes/No). Univariate analyses were first performed on each of the studied factors to select candidate independent variables for the multivariable analysis. Those factors with a *P* value <0.25 in the univariate analyses^26^ were selected as candidate variables for stepwise multivariable logistic regression to delineate factors independently associated with the screening behavior outcome. The results of significant factors identified were presented with their odds ratio (OR) and 95% confidence intervals (CI). All statistical tests involved were two-tailed and statistical significant level was set at 0.05.

**Table 1:** Demographic characteristics and health status of the respondents (*n*=1,002)

Characteristics	*n* (%)
Age*, yrs	
50-59	382 (38.1)
60-69	273 (27.2)
70-79	251 (25.0)
80 or above	96 (9.6)
Education level	
Primary or below	400 (40.1)
Secondary	417 (41.8)
Matriculation or above	181 (18.1)
Employment status	
Not employed	671 (67.2)
Employed	327 (32.8)
Monthly household income (HK$)	
<10,000	324 (32.8)
10,000-29,999	226 (22.9)
≥30,000	130 (13.2)
Don’t know/decline to disclose	308 (31.2)
Marital status	
Single/divorced/widowed	188 (18.9)
Married/cohabited	806 (81.1)
Family history of cancer	
No/don’t know	785 (78.3)
Yes	217 (21.7)
Health status	
Chronic illness	
Any confirmed chronic illness	439 (43.8)
Serious disease	
Ever had a serious disease or cancer	86 (8.6)
Smoking status	
Never smoker	568 (56.7)
Ex-smoker	230 (23.0)
Current smoker	204 (20.4)

Data are presented as frequency (%). *Age distribution of the whole men population aged 50 or above in Hong Kong in 2011: aged 50-59, 0.565 million (46.8%); aged 60-69, 0.326 million (27.0%); aged 70-79, 0.213 million (17.7%); aged 80 or above, 0.103 million (8.5%).

**Table 2:** Perceived health status and utilization of complementary therapies

Characteristics	*n* (%)
Health related perceptions	
Perceived health status	
Excellent/very good/good	489 (48.8)
Fair/poor	513 (51.2)
Perceived that following practices are good for health	
Doing exercise	754 (75.2)
Maintaining a healthy diet	666 (66.5)
Visiting a doctor regularly	422 (42.1)
Visiting a Chinese herbalist regularly	183 (18.3)
Taking dietary supplements	154 (15.4)
Perceived susceptibility to cancer (ranged from 1= not at all likely to 10= extremely likely)	
5	676 (67.5)
>5	80 (8.0)
Unsure	246 (24.6)
Utilization of complementary therapy	
Ever used the following complementary therapies	
Acupuncture	117 (11.7)
Cupping	97 (9.7)
Chinese herbal medicine	282 (28.1)
Bone setting	250 (25.0)
Chinese massage	141 (14.1)
Use of complementary therapy index	
0 (≤50^th^ percentile)	547 (54.6)
1-2 (>50^th^-75^th^ percentile)	208 (20.8)
3 (>75^th^ percentile)	247 (24.7)

Data are presented as frequency (%).

## Results

A total of 1,002 men aged 50 or above completed the anonymous survey and were included in the study (response rate =67%). The age distribution of the sample collected was reasonably comparable to that of the local population, although the 50-59 age group was somewhat under-represented (footnote to **Table 1**)^27^.

### Demographic characteristics and health status

The demographic characteristics and health status of the respondents are shown in **Table 1**. The mean age of the respondents was 64.2±10.2 years and ranged from 50 to 94 years. More than half had received a secondary education or above (60%). The majority were married or cohabiting (81%) and unemployed (67%), with 36% reporting a middle (HK$10,000-29,999, 1US$≈7.8HK$) or high (HK$30,000+) monthly household income, although a considerable proportion (31%) did not know or declined to disclose their income. Less than half had at least one type of chronic illness (44%), and only 9% had ever had cancer or other serious disease. One fifth of the men were current smokers, and 22% had a family history of cancer.

### Perceived health status and use of complementary therapies

The respondents’ perceived health status and utilization of complementary therapies are shown in **Table 2**. Slightly over half reported their health status as fair or poor (51%), and the majority believed that doing exercise (75%) and maintaining a healthy diet (67%) were good for their health. Fewer than half perceived that visiting a doctor regularly was good for the health (42%), and only a small proportion believed that visiting a Chinese herbalist regularly (18%) or taking dietary supplements (15%) were good for the health either. More than two-third (68%) perceived that they were unlikely susceptible to cancer.

Among the five most commonly used complementary therapies in Chinese societies^28^, it was interesting to note that the usage rates (‘had ever used’) reported by this sample were not high: acupuncture (12%), cupping (10%), herbal medicine (28%), bone setting (25%) and Chinese massage or ‘tuina’ (14%).

### Prostate cancer screening behavior

Among all respondents, only 10% confirmed that they had ever had a PSA test. The three main reasons for having the most recent PSA test were: (1) regular physical check-ups (39%); (2) prompted by local signs and symptoms (34%) and (3) physician’s recommendation (21%). The three most common reasons cited by respondents for never having had a PSA test were: (1) they did not think it was necessary (44%); (2) they did not know it was available (33%) and (3) they regarded themselves as healthy all along (8%) (**Table 3**). Only 4% reported that they had been recommended by health professionals for a PSA test.

**Table 3:** Prostate cancer screening behaviour—prostate-specific antigen (PSA) test

Characteristics	*n* (%)
Any health professional recommended a PSA test	
No/unsure	965 (96.3)
Yes	37 (3.7)
Ever had a PSA test	
No	898 (89.6)
Yes	95 (9.5)
Unsure	9 (0.9)
Among those who ever had a PSA test (*n*=95)	
Time since the most recent test	
<1 year	27 (28.4)
1-2 years	28 (29.5)
3-4 years	21 (22.1)
5-6 years	7 (7.4)
>6 years	7 (7.4)
Can’t remember	5 (5.3)
Ever had an abnormal test result	
No	81 (85.3)
Yes	14 (14.7)
The three main reasons for the most recent test	
Body checkup	37 (38.9)
Prompted by local signs and symptoms*	32 (33.7)
Doctor suggestion	20 (21.1)
Among those who have not the test (*n*=898)	
The three most important reasons for not having the test	
Not necessary	399 (44.4)
Don’t know this test is available	296 (33.0)
Healthy all along	69 (7.7)

Data are presented as frequency (%). *Difficult in micturition, frequent micturition, have pain, lumps or bleeding.

### Factors associated with having had a PSA test

**Table 4** shows the results of factors that were associated with having had a PSA test. Stepwise multivariable logistic regression, with removal and entry criteria set at *P*>0.1 and *P*<0.05 respectively, using those factors with *P* values <0.25 in univariate analysis as candidate independent variables revealed that employment status, use of complementary therapy, visiting a doctor regularly seen as good for health and recommendation from health professional were all significantly associated with ever having had a PSA test (**Table 4**). Respondents who were employed were less likely to have a PSA test [employed *vs*. unemployed, OR=0.4 (95% CI: 0.2-0.8), *P*=0.004]. Those respondents with most frequent use of complementary therapies were more likely to have a PSA test than those who had never used them [OR=2.1 (1.2-3.6), *P*=0.006]. Perceptions of visiting a doctor as good for health were associated with increased odds of ever having had a PSA test [OR=2.7 (1.7-4.4), *P*<0.001]. Finally, and remarkably, recommendations from health professionals had a very strong influence on the screening test [OR=25.9 (11.8-56.7), *P*<0.001].

**Table 4:** Factors associated with ever having had a PSA test

	Ever had a PSA test, *n* (%)		OR_U_	*P*	OR_A_ (95% CI)	*P*
	No (*n*=898) (%)	Yes (*n*=95) (%)				
Demographic characteristics						
Age, yrs						
50-59 (ref)	358 (94.2)	22 (5.8)	1		NS	
60-69	243 (89.3)	29 (10.7)	1.94	0.024		
70-79	213 (86.2)	34 (13.8)	2.60	0.001		
80 or above	84 (89.4)	10 (10.6)	1.94	0.098		
Education level						
Primary or below (ref)	362 (91.6)	33 (8.4)	1		NS	
Secondary	374 (90.1)	41 (9.9)	1.20	0.452		
Matriculation or above	158 (88.3)	21 (11.7)	1.46	0.201		
Employment status						
Not employed (ref)	587 (88.4)	77 (11.6)	1		1	
Employed	307 (94.5)	18 (5.5)	0.45	0.003	0.41 (0.22-0.75)	0.004
Monthly household income (HK$)						
<10,000 (ref)	280 (87.2)	41 (12.8)	1		NS	
10,000-29,999	207 (92.4)	17 (7.6)	0.56	0.056		
≥30,000	116 (89.9)	13 (10.1)	0.77	0.427		
Don’t know/decline to disclose	282 (92.5)	23 (7.5)	0.56	0.033		
Marital status						
Single/divorced/widowed (ref)	170 (91.4)	16 (8.6)	1		NE	
Married/cohabited	721 (90.2)	78 (9.8)	1.15	0.628		
Family history of cancer						
No/don’t know (ref)	712 (91.5)	66 (8.5)	1		NS	
Yes	186 (86.5)	29 (13.5)	1.68	0.029		
Health status						
Any confirmed chronic illness?						
No (ref)	515 (92.5)	42 (7.5)	1		NS	
Yes	383 (87.8)	53 (12.2)	1.70	0.015		
Ever had a serious disease or cancer?						
No (ref)	828 (91.2)	80 (8.8)	1		NS	
Yes	70 (82.4)	15 (17.6)	2.22	0.010		
Smoking status						
Never smoker (ref)	499 (88.8)	63 (11.2)	1		NS	
Ex-smoker/current smoker	399 (92.6)	32 (7.4)	0.64	0.046		
Use of complementary therapy						
Use of complementary therapy index						
0 (≤50^th^ percentile)	499 (92.1)	43 (7.9)	1		1	
1-2 (>50^th^-75^th^ percentile)	189 (91.7)	17 (8.3)	1.04	0.886	0.86 (0.44-1.68)	0.660
≥3 (>75^th^ percentile)	210 (85.7)	35 (14.3)	1.93	0.006	2.11 (1.24-3.57)	0.006
Recommendation from health professional						
Any health professional recommended the test						
No/unsure (ref)	886 (92.6)	71 (7.4)	1		1	
Yes	12 (33.3)	24 (66.7)	25.0	<0.001	25.9 (11.8-56.7)	<0.001
Health related perceptions						
Perceived health status						
Excellent/very good/good (ref)	449 (92.4)	37 (7.6)	1		NS	
Fair/poor	449 (88.6)	58 (11.4)	1.57	0.042		
Perceived doing exercise is good for health						
No (ref)	231 (93.9)	15 (6.1)	1		NS	
Yes	667 (89.3)	80 (10.7)	1.85	0.035		
Perceived maintaining a healthy diet is good for health						
No (ref)	309 (93.1)	23 (6.9)	1		NS	
Yes	589 (89.1)	72 (10.9)	1.64	0.047		
Perceived visiting a doctor regularly is good for health						
No (ref)	544 (94.6)	31 (5.4)	1		1	
Yes	354 (84.7)	64 (15.3)	3.17	<0.001	2.68 (1.65-4.38)	<0.001
Perceived visiting a Chinese herbalist regularly is good for health						
No (ref)	743 (91.4)	70 (8.6)	1		NS	
Yes	155 (86.1)	25 (13.9)	1.71	0.031		
Perceived taking dietary supplements is good for health						
No (ref)	768 (91.3)	73 (8.7)	1		NS	
Yes	130 (85.5)	22 (14.5)	1.78	0.027		
Perceived susceptibility to cancer (ranged from 1=not at all likely to 10=extremely likely)						
≤5 (ref)	614 (91.6)	56 (8.4)	1		NS	
>5	68 (85.0)	12 (15.0)	1.93	0.054		
Unsure	216 (88.9)	27 (11.1)	1.37	0.202		

ref, Reference group of the categorical variable; OR_U_, univariate odds ratio; OR_A_, odds ratio adjusted for other significant factors obtained from stepwise logistic regression analysis using variables with *P* value <0.25 in univariate analysis as candidate variables; NS, not statistically significant in multivariate analysis; NE, not entered into multivariable analysis; PSA, prostate-specific antigen.

## Discussion

### Uptake of PSA testing

The uptake rate of PSA testing was 10%, lower than in other studies conducted in comparable groups^17^^,^^18^. The three main reasons for having the test were ‘regular physical check-ups’, ‘presence of unusual signs and symptoms’, and ‘doctor’s recommendation’. On the other hand, respondents had not undergone the test because they perceived the test was ‘not necessary’, they ‘did not know the test was available’ or they considered they were ‘healthy all along’. The findings reflect the respondents lacked knowledge and had misconceptions about prostate cancer and the benefits of screening. Similar results have also been reported by other studies, indicating that a lack of knowledge and faulty perceptions of prostate cancer risk influence males in adopting certain health behavior^17^^,^^18^^,^^29^.

Another possible reason may be insufficient health promotion and education of the public—only 3.7% (*n*=37) of the respondents received recommendations from health professionals for a PSA test. In fact, the Cancer Expert Working Group on Cancer Prevention and Screening^30^ concluded that insufficient scientific evidence was available to recommend whether or not prostate cancer screening in asymptomatic men should be carried out. Men are therefore encouraged to discuss with their doctors the benefits and risks of such screening. However, lack of knowledge about prostate cancer in men and insufficient health promotion of prostate cancer screening may hinder men’s awareness and compliance of the recommendation.

### Factors associated with PSA testing

Respondents more likely to have had a PSA test were those who were unemployed, used complementary therapies for health promotion and restoration, perceived visiting a doctor regularly as good for health or had received a recommendation from health professionals. It was interesting to find that unemployed respondents were more likely to take a PSA test. Perhaps employment status may be directly or indirectly related to age. The results of univariate analysis showed that respondents aged 50-59 were less likely to take a PSA test than those aged 60-79, though the result was not significant in multivariate analysis. Further studies are needed to get a clearer picture of the perceptions and attitudes towards having a PSA test among older adults who are or are not employed.

The use of complementary therapy was another associated factor identified in multivariate analysis. In Chinese societies, various complementary therapies are commonly used to promote and restore health, which may be why those who have resorted to such complementary measures are more receptive to undergoing a PSA test.

Regarding regular visits to a doctor as good for health and recommendations from health professionals were two important factors associated with the uptake of PSA testing. In particular, the odds of test uptake were 25.9 times better among participants who had received recommendations from health professionals than among those who had not. Similar results have been produced by other studies conducted with comparable age groups^17^^,^^24^. Health professionals should therefore take up a significant role in health promotion and education to increase awareness of cancer prevention and detection among the male population in Hong Kong. They would also be expected to explain and discuss the potential benefits and risks of PSA testing to help their patients come to a decision.

## Limitations

There are several limitations to this study. First, it uses a cross-sectional survey design, which makes it difficult to establish the causality and identify the predictors of PSA testing uptake. Second, other factors that may be associated with the uptake, such as a history of benign prostate hypertrophy or a family history of prostate cancer, were not included. Third, recall bias exists in self-report data. Last, but not the least important, data collected from a self-report survey, such as questions about having ever had a PSA test or being recommended by a health professional, were not validated.

## Conclusion

This study reported the uptake rate of PSA testing, which was very low, and identified associated factors among older Chinese men. After adjusting for confounding variables, those respondents who were not employed, used complementary therapy, perceived visiting a doctor regularly as good for health and had been recommended by a health professional were found to be more likely to have a PSA test. Among all the factors identified, recommendations from health professionals showed the strongest association with PSA test uptake in this population. Health professionals should take an active role in educating this particular population in cancer prevention and detection.
